# Troponin-I enhances and is required for oncogenic overgrowth

**DOI:** 10.18632/oncotarget.10616

**Published:** 2016-07-15

**Authors:** Sergio Casas-Tintó, Antonio Maraver, Manuel Serrano, Alberto Ferrús

**Affiliations:** ^1^ Instituto Cajal, C.S.I.C., Madrid, Spain; ^2^ Centro Nacional de Investigaciones Oncológicas (CNIO), Madrid, Spain; ^3^ Institut de Recherche en Cancérologie de Montpellier (IRCM - U1194), Inserm and Université de Montpellier, ICM, Montpellier, France

**Keywords:** cell proliferation, Drosophila, cell competition, cancer

## Abstract

Human tumors of various tissue origins show an intriguing over-expression of genes not considered oncogenes, such as that encoding Troponin-I (TnI), a well-known muscle protein. Out of the three TnI genes known in humans, the slow form, *TNNI1*, is affected the most. *Drosophila* has only one TnI gene, *wupA*. Here, we studied excess- and loss-of function of *wupA* in *Drosophila*, and assayed *TNNI1* down regulation in human tumors growing in mice. *Drosophila* TnI excess-of-function increases proliferation and potentiates oncogenic mutations in *Ras, Notch* and *Lgl* genes. By contrast, TnI loss-of-function reduces proliferation and antagonizes the overgrowth due to these oncogenic mutations. Troponin-I defective cells undergo *Flower*- and *Sparc*-dependent cell competition. TnI can localize to the nucleus and its excess elicits transcriptional up-regulation of *InR, Rap1* and *Dilp8*, which is consistent with the increased cell proliferation. Human tumor cell lines treated with a human Troponin-I peptide arrest in G_0_/G_1._ In addition, proliferation of non-small-cell lung carcinoma xenografts in mice is restrained by *TNNI1* down-regulation. Thus, Troponin-I reveals a novel function in cell proliferation that may be of therapeutic interest in certain types of cancer.

## INTRODUCTION

Troponin-I (TnI) binds F-actin to regulate muscle contraction through a complex with Troponin-C, Troponin-T and Tropomyosin which decorates the thin filaments of the sarcomere at regular intervals [1-3]. However, the unexpected discovery of TnI in non-muscle cells [4] raised questions about its role in general cell biology. We showed previously that *Drosophila* TnI expresses early in the syncytial embryo before cell type specification. Also, TnI in S2 cell cultures traffics between the nucleus and the cytoplasm using a sumoylation-dependent mechanism [4]. Vertebrate TnI had been detected in cartilage as an anti-angiogenic factor, which, indirectly, would prevent metastatic liver growth in a mouse model of pancreatic primary tumor [5-6]. In spite of these observations, however, the role of TnI outside the well characterized muscle cells had remained enigmatic.

The classical concept of “cancerization field” [7] has often been linked to cell competition [8-10], a term originally coined in *Drosophila* to describe the physical elimination of unfit cells due to their slower mitotic rate with respect to surrounding neighbors [11]. The phenomenon involves the activation of caspase 3 [12], the JAK-STAT system [13-14] perhaps independently from Myc [15] although Myc seems involved in cell competition in *Drosophila* [12, 16] as well as in mammals [17-18]. Data in *Drosophila* show that winners in cell competition elicited by a variety of mechanisms (*Minute, Myc, tkv, scrib*, etc.) recognize adjacent losers by a so-called Flower code [8, 19-20], which drives cells to apoptosis, eventual extrusion and ultimate sequestration of their debris by attracted macrophage-like hemocytes [21]. Although cell competition is not always a pre-condition for a cell to become cancerous, the association is frequent enough as to justify the study of this phenomenon in the *Drosophila* models of human cancers in the context of TnI expression changes. Thus, we set out to characterize the effects of excess and depletion of TnI in *Drosophila* epithelial cells of the wing and eye discs, concomitant with the expression of mutant forms of classical oncogenes. In addition, we validated the fly results in mammals by analyzing the effects of down-expression of human TNNI1 in tumor growth of human xenografts in mice.

## RESULTS

### Human troponin I in cancer databases

**Figure 1:**
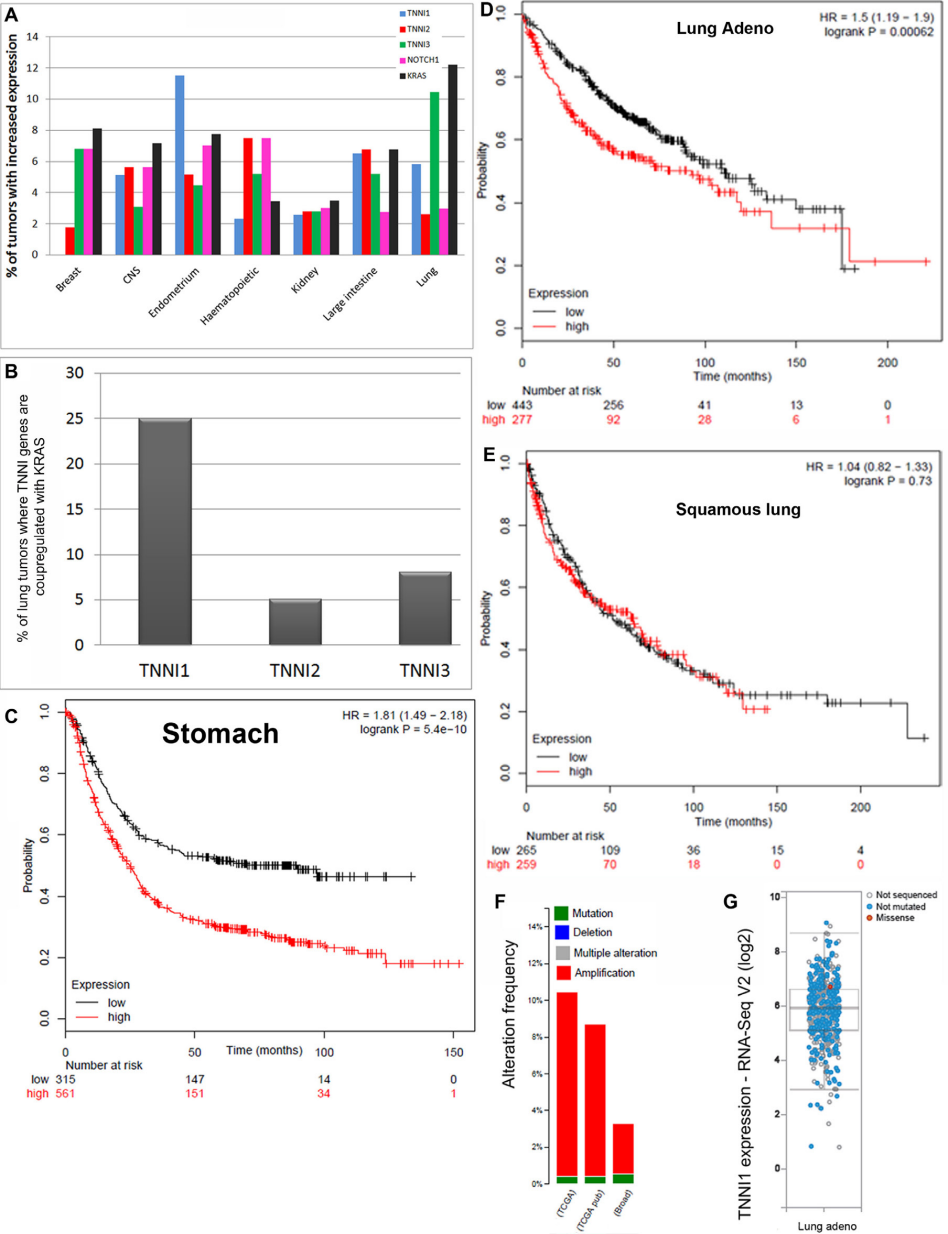
Association data in cancer databases **A.** Percentage of human tumors reported to have at least 2 fold increased expression of *TNNI1, TNNI2, TNNI3, KRas* or *Notch* genes in different organs and tissues. Note that *TNNI1* and *KRas* are simultaneously over-expressed in endometrium, large intestine and lung cancers mainly. **B.** Percentage of lung cancer cases where *TNNI* and *KRas* genes are co-upregulated. Note that *TNNI1*over-expression*,* but not *TNNI2* or *TNNI3,* is associated with *KRas* over-expression. The choice of lung samples in this comparison is justified because most cell lines available for this study originate from this organ. Data in A-B are from COSMIC database. **C.**, **D.** The Kaplan-Meier data plot show that stomach (C) and, to a lesser extent, lung adenocarcinoma (D) life expectancy of patients correlates inversely with the high expression of TNNI1 (red). **E.** One exception to this correlation, however, is the case of squamous lung cancer. **F.**, **G.** A third database, CBIOPORTAL, also shows that high expression of *TNNI1*, either through gene copy amplification (F) or expression by RNA seq (G), are frequent in lung adenocarcinoma.

Following the discovery that fly TnI is expressed in virtually all cell types, we explored the possible involvement of the human homologue in pathology. We began with cancer as reported in databases. We noticed a significant proportion of cases with altered expression of Troponin-I (*TNNI*) genes in the Catalogue of Somatic Mutations in Cancer (http://cancer.sanger.ac.uk/cosmic), particularly *TNNI1* in lung, ovary and endometrium tumors, among others (Figure 1A, 1B). Also, the Kaplan-Meier data plot (http://kmplot.com/analysis/) [22] shows that high levels of *TNNI1* are a bad prognosis indicator in stomach cancer and in lung adenocarcinoma (Figure 1C-1E). Finally, a third database (http://www.cbioportal.org) show that lung adenocarcinoma often exhibits *TNNI1* over-expression, either through gene amplification or gene expression changes (Figure 1F, 1G). Thus, out of the three TnI genes known in humans, the slow form, *TNNI1*, seems affected the most. In addition, *TNNI1* is the closest sequence homologue to the single *Drosophila* TnI encoding gene, *wupA*. The association of *TNNI1* excess with tumor type, however, does not seem absolute and a notable exception is the squamous lung cancer (Figure 1E). The correlations between *TNNI1* expression levels and cancer type in these databases justify an experimental study to identify the underlying biology.

### TnI over-expression exacerbates oncogenic mutant phenotypes and elicits transcriptional changes

**Figure 2:**
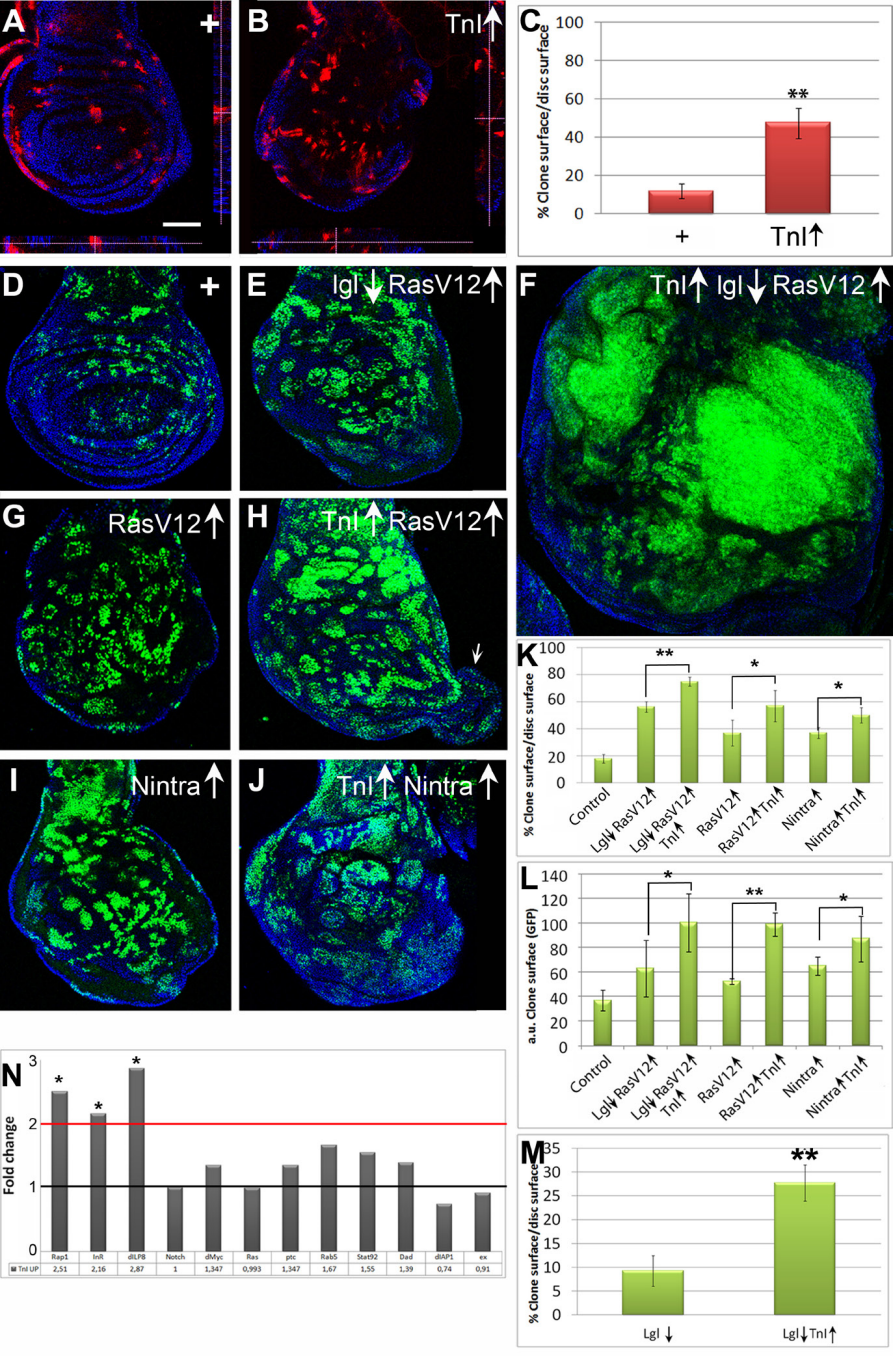
TnI exacerbates oncogene overgrowths **A.**, **B.** Wing discs with FLP-out clones of *UAS-LacZ* (+) (A) or *UAS-TnI* (TnI↑) (B) genotypes (♀ *x/hs-FLP*; +/+; *actin-FRT-Stop-FRT-Gal4,UAS-myrRFP/+*). Insets (dotted lines) show Z-axis views of (A) and (B). Note that over-expressing TnI cells (red) are not extruded from the epithelium. **C.** Quantification of data from experiments shown in A and B. Note the larger extent of TnI over-expressing clones. **D.**-**F.** Normal and experimental FLP-out clones showing the synergy between TnI and oncogenic mutants. Genotypes: (D = ♀ *+/hs-FLP*; *actin-FRT-Stop-FRT-Gal4,UAS-GFP/+*), (E = ♀ *+/hs-FLP*; *actin-FRT-Stop-FRT-Gal4,UAS-GFP/UAS-RasV12; UAS-lgl/+*) and (F =♀ *UAS-TnI / hs-FLP*; *actin-FRT-Stop-FRT-Gal4,UAS-GFP/UAS-RasV12; UAS-lgl/+*). Note the massive overgrowth in the last genotype (F). All images were taken at the same magnification and laser parameters. **G.**, **H.** Equivalent clones (same *Gal4* driver cassette) using *Ras* alone. The potentiation effect is still evident and includes disc protrusions (arrow in H). **I.**, **J.** Clones using the constitutively active form of *Notch*, *N*. Genotypes: (I =♀ *+/hs-FLP*; *UAS-N*/*actin-FRT-Stop-FRT-Gal4,UAS-GFP; +/+*) (J = ♀ *UAS-TnI / hs-FLP*; *UAS-N*/*actin-FRT-Stop-FRT-Gal4,UAS-GFP; +/+*). **K.**, **L.** Relative and absolute quantifications of wing disc mosaics. Note the synergy in phenotypes that combine TnI↑ with oncogenes. **M.** Relative quantification of wing disc *lgl*clones. Scale bar in A = 50μm in A-J. Confocal images are maximal projections. **N.** Transcriptional effect on twelve genes as indicated by qRT-PCR assays. Genotype: *♂ UAS-LacZ/tub-Gal80; tub-Gal4LL7/+* (Control) ♂ *UAS-TnI ; tub-Gal80 /+; tub-Gal4LL7/+*. Fold change values are indicated below each gene symbol. Note the significant increase of *Rap1, InR* and *dILP8* expression while all others, including *Myc, Ras* and *Notch*, remain unchanged.

For brevity, over- and down-expresser constructs are indicated by up and down arrows, respectively, adjacent to the gene symbol. We evaluated the functional relevance of TnI over-expression in *Drosophila* by means of FLP-out mosaics using a construct based in the driver *actin-Gal4* (see Mat. and Methods). The effectiveness of the TnI over-expressing construct was validated by qRT-PCR assays (Figure S1A).Wing disc clones over-expressing TnI were, on average, three times larger than controls (Figure 2A-2C) due to increase in the number of cells per clone rather than to cell size (Figure S1B, C), indicating that TnI promotes proliferation.

To expand on this initial observation, mosaics were generated in standard oncogenic mutant backgrounds. As currently understood, a wing cell deficient for the polarity protein Lethal-giant-larvae (Lgl) will be eliminated [23-25]. However, if combined with a constitutively active form of Ras, Ras^V12^, it will proliferate yielding a tumor instead of being eliminated. This escape from elimination is thought to be due to clone cells reaching a critical mass in which self-protecting factors would allow overgrowth, albeit without the correct tissue arrangement, hence yielding a tumor [26].

In the case of *Ras^V12^/lgl↓*, the simultaneous excess of TnI yields massive overgrowths as compared to those produced by the *Ras^V12^/lgl↓* combination alone (Figure 2D-2F, 2K, 2L). Larvae with this type of clones, induced in late LII stage, never reach adulthood. The phenotype was noticeable also in *Ras^V12^* only background, including the generation of overgrowths that protrude from the normal wing disc in about 40% of cases (see arrow in Figure 2H), albeit with a less extreme phenotype than in the double mutant combination (Figure 2G, 2H, 2K, 2L). Likewise, the exacerbated phenotype was also evident in the *lgl↓* only background (Figure 2M). These larvae also fail to reach adulthood. In the case of the constitutively active Notch, *N^intra^*, the potentiation effect is strong (Figure 2I-2L) but occasional adults are recovered with visible overgrowths composed of an undefined cell type (Figure S1D-F).

A plausible mechanism to explain how an excess of TnI could increase cell proliferation remained to be identified. To address this issue and since we had previously demonstrated that TnI can translocate to the nucleus [4], we considered the possibility that the excess of TnI could trigger transcriptional changes in target genes, as some classical oncogenes do (e.g.: *Ras*, [27-28]). We assayed by qRT-PCR the transcription of a limited set of 12 genes selected by their reported involvement in cellular events affected in the TnI mosaics above. To prevent potential developmental effects, we used a genotype that allows the temporal control of generalized TnI over-expression (♂ *tubLL7-Gal4>UAS-TnI; Gal80^ts^/+*). Using a temperature shift to inactivate the Gal80 repressor, we triggered the driver expression at day 5 of adulthood and processed whole body samples 36 h later. This schedule aimed to capture the early transcriptional changes that follow TnI over-expression, although the magnitude of the eventual changes will not be expected to be great. In spite of this caveat, the data show over two fold increased transcription of three genes known to be involved in growth rate control: the insulin receptor (*InR*) [29] the Ras-related small GTPase protein 1 (*Rap1*) [30] and the insulin-like peptide 8 (*Dilp8*) [31-32] (Figure 2N). These changes are consistent with the increased proliferation observed in TnI over-expression clones (Figure 2A-2C). For example, Dilp8 peptide is autonomously produced by imaginal disc cells undergoing tumoral growth [31]. Also, Rap1 mediates the cell transition from adhesion to migration in tumoral growth [30] through the activation of ERK and it activates Raf in conjunction with Ras [33-34]. As for InR, its role in organ size and cell proliferation control is widely documented. Interestingly, *Notch, Ras* and *Myc* genes are not transcriptionally affected by the excess of TnI (Figure 2N) which suggests that the gene expression changes elicited by TnI and those elicited by these oncogenes are different (see Discussion). Although the number of target genes analyzed here is limited, it is evident that, akin to classical oncogenes, the excess of TnI causes changes in transcription that are consistent with the observed cellular effects (see Discussion for possible additional components of the TnI related transcriptional mechanism).

### TnI under-expression reduces cell proliferation and triggers cell competition

Next, we tackled the cellular effects of TnI depletion. To monitor TnI we used the previously reported J4 antibody and generated a monoclonal antibody, SC1. SC1 identifies TnI in its canonical position in muscle sarcomeres (Figure S2A-C). In addition, SC1 specificity was validated in large Minute^+^ FLP-out clones for the null mutant *TnI^23437^*in the wing disc (Figure S2D, E). The TnI immunosignal is also evident in the eye disc behind the morphogenetic furrow where cells in mitosis are synchronized (Figure S2F, G). TnI appears cytoplasmic in the anterior proliferating domain but it accumulates in the nucleus at the time of the second mitotic wave (SMW).

To analyze the TnI loss-of-function phenotypes, we used null mutations or TnI^RNAi^. The RNAi construct was validated as a strong attenuator of TnI expression in the wing pouch domain (*rnGal4>UAS-TnI^RNAi^*) (Figure S3A, B). Wing FLP-out clones of TnI^RNAi^ expressing cells show reduced proliferation (Figure S3C, D, F). The effect is counterbalanced by the co-over expression of TnI (Figure S3E, F) demonstrating that both effects, over- and under-proliferation, are caused by the same protein, TnI. In wing discs, at 48h after clone induction, clones contain 1-4 cells only (Figure S4A), and by 72h virtually all TnI mutant cells have been eliminated (Figure S4B). This elimination can be suppressed by the simultaneous overexpression of TnI (Figure S3G, H). Deficits in proliferation are also evident in FLP-out clones using the null mutation *TnI^23437^* in GFP-tagged twin configuration (Figure S3I-K). Comparison of 48 *versus* 72 h clones shows that *TnI^23437^* homozygous cells are eliminated (Figure S3L-N).Thus, the null mutation and the RNAi yield the same phenotype (see also below). The longer survival of *TnI^23437^ versus* TnI^RNAi^ cells can be explained by the difference in the two mechanisms to abolish gene expression. Upon the generation of the clone, the TnI^RNAi^ cell suffers interference in mRNA translation while the *TnI^23437^* cell still can undergo translation of existing mRNA molecules. Actually, immunostaining of *TnI^23437^* cells still shows traces of the protein (Figure S3J). Thus, TnI deficient cells can undergo 1-2 mitosis, likely because of perdurance of native TnI from the mother cell, but their inefficient proliferation leads to their final elimination. A similar phenomenon of slight differences in the mutant *versus* RNAi phenotypes has been reported also for the gene *Torso* [35].

**Figure 3:**
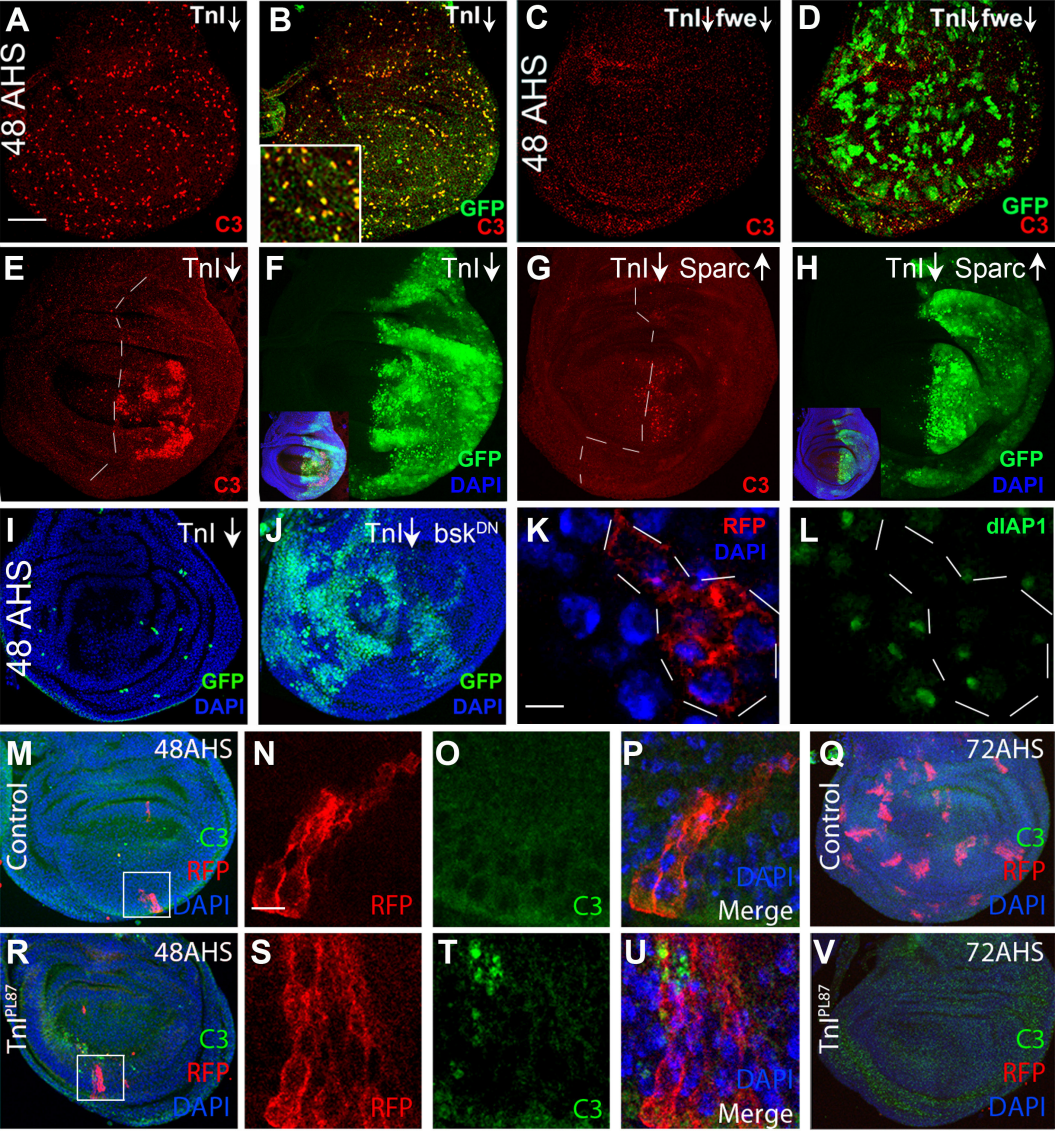
Cell death mechanism by TnI deficiency **A.**, **B.** GFP marked FLP-out clones at 48 h after induction by heat shock (48AHS) express activated caspase-3 (C3, red). Note the coincidence of GFP and C3 signals in inset in B. **C.**, **D.** The simultaneous down-regulation of Flower (*fwe*) allows growth of TnI deficient clones and suppress C3 activation (genotype: ♀ *+/hs-FLP* ; *actin-FRT-Stop-FRT-Gal4,UAS-GFP/UAS-fwe* ; *UAS-TnI*/+). **E.**, **F.** The same effect is detected when the entire posterior wing compartment (*en-Gal4*) expresses the *TnI*(TnI↓). See inset in F as a whole disc reference. Genotype: *+; enGal4,UAS-GFP/+ ; UAS-TnI/+*. **G.**, **H.** The simultaneous up-regulation of the cell defense factor Sparc also suppresses the C3 activation. Genotype: + ; *enGal4,UAS-GFP/UAS-Sparc ; UAS-TnI/+*. Dotted lines indicate compartment border. See inset in H for whole disc reference. **I.**, **J.** TnI-dependent cell death in 48 h AHS FLP-out clones is suppressed by the simultaneous down-regulation of bsk/JNK using a dominant negative form. Genotype: *UAS-bsk/hs-FLP* ; +/+ ; *actin-FRT-Stop-FRT-Gal4,UAS-GFP/UAS-TnI*. **K.**, **L.** TnI deficient cells (RFP, dotted line marks the clone) switch off the expression of the survival factor dIAP1 reported by GFP in most cells (L, green). Genotype: *hs-FLP ; actin-FRT-Stop-FRT-Gal4,UAS-RFP/+; dIAP1-GFP/UAS-TnI*. **M.**-**Q.** Control clones at 48h AHS do not exhibit activated caspase 3 and maintain growth by 72 h AHS (Q). Inset in M is magnified in N. Genotype: ♀ *y w TnI/+; UAS-myr-RFP/hs-FLP38; tubGal4 • FRT82B Gal80 Dp(1;3)JC153/FRT82B.* **R.**-**V.** Null *TnI* wing disc clones show activated caspase 3 and are eliminated by 72 h AHS (V). Inset in R is magnified in S. Genotype: ♂ *y w TnI; UAS-myr-RFP/hs-FLP38 ; tubGal4 • FRT82B Gal80 Dp(1;3)JC153/FRT82B.* Images are stacks of several confocal planes. Bar in A = 50 μm (A-J, M, Q, R and V). Bar in K = 5 μm (K, L, N-P and S-U).

To further analyze the cell elimination process, we reduced simultaneously Flower (*fwe*), a specific label of unfit cells, or increased the levels of Dpp, a survival factor for loser cells in cell competition. Both procedures largely rescue the TnI deficient cells from elimination allowing them to proliferate further (Figure S4C-F). The process elicited by TnI loss includes activation of Caspase 3 (C3) (Figure 3A, 3B) which is suppressed to a large extent by the co-down-regulation of *fwe* (Figure 3C, 3D). This feature is also evident when an entire wing compartment (*engrailed-Gal4*) is made TnI deficient (Figure 3E, 3F). C3 activation is also largely suppressed by the up-regulation of Sparc, another specific cell defense signal during cell competition [36] (Figure 3G, 3H and Figure S3O-Q). The unstable nature of the equilibrium between the cell death triggered by TnI depletion, and the cell survival promoted by the excess of Sparc may account for the residual cell death revealed as C3 positive spots (Figure 3G; Figure S3O). The down-regulation of bsk/JNK, a signal needed for the elimination of cells during cell competition [37], is another effective procedure to prevent TnI^RNAi^ expressing cells from C3 activation in FLP-out clones (Figure 3I, 3J). As expected, TnI deficient cells switch-off the pro-survival signal DIAP-I [38] (Figure 3K, 3L). As further evidence that RNAi and null mutants yield the same phenotypes, we analyzed mitotic recombination clones of *PL87*, another null mutation that, as *23437* is a rearrangement of the regulatory region of the *wupA* gene [39]. These PL87 clones activate C3 at 48 h AHS (Figure 3M-3P, 3R-3U) and are eliminated by 72 h AHS (Figure 3Q, 3V). Together, these features indicate that the elimination of TnI deficient cells occurs through a mechanism that includes apoptosis, and it is akin to cell competition [12, 16].

### Tumor overgrowths require TnI

**Figure 4:**
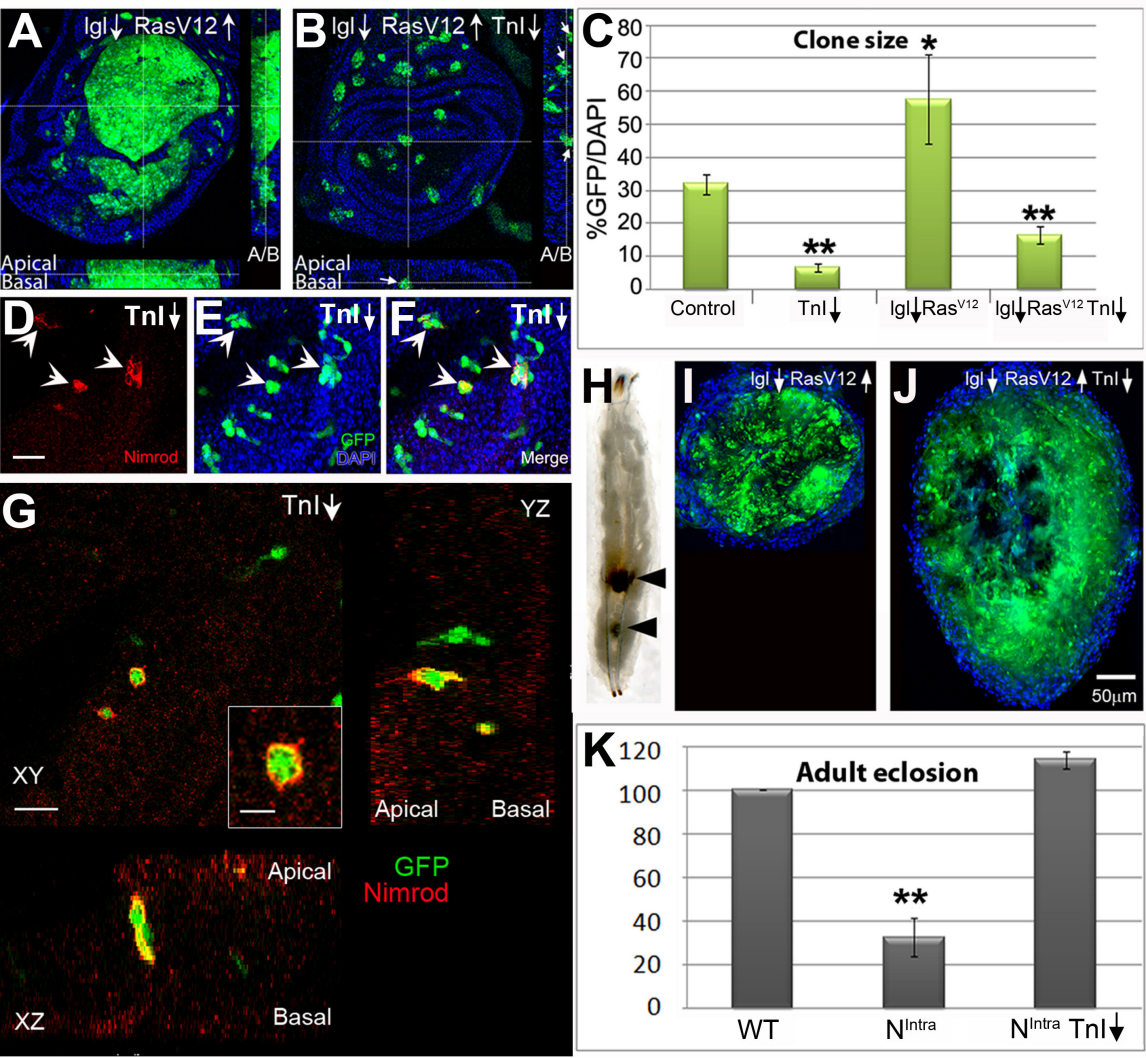
TnI down-regulation triggers cell death and suppresses tumor overgrowth **A.**, **B.** Over-proliferating *lgl↓/Ras* cells in FLP-out wing disc clones are eliminated, possibly through basal extrusion, when TnI is down-regulated. Insets (dotted lines) show Z-axis views with the apico-basal (A/B) localization of GFP marked cells expressing the three constructs. Most of these cells locate basally (arrows). Genotype: *hs-FLP/+ ; actin-FRT-Stop-FRT-Gal4,UAS-GFP/UAS-Ras; UAS-lgl/ UAS-TnI*. **C.** Quantification of data from experiments shown in A, B and E and from a control genotype (*+/hs-FLP* ; *actin-FRT-Stop-FRT-Gal4,UAS-GFP/UAS-LacZ*). **D.**-**F.** Extruded TnI deficient cells from wing discs are captured by Nimrod-positive hemocytes immune-labelled in red (arrows). **G.** High magnification views in the three visual planes to demonstrate that Nimrod-positive hemocytes engulf the GFP marked TnI^RNAi^ expressing cells. **H.**-**J.** Extruded GFP-tagged cells expressing oncogenic genotypes often accumulate as one or more neoplasic inclusions in the larval fat body. The neoplasic body grows larger when TnI is down-regulated, possibly reflecting the larger number of extruded cells (J). Note that images in I and J are at the same magnification (see scale in J). **K.** Adult survival effects due to *N* expression in the *gmr-Gal4* domain, and their suppression by the simultaneous TnI down-regulation. Genotype: *Canton-S* (WT) and *gmr-Gal4/UAS-N; UAS-TnI/+*. Images are stacks of several confocal planes. Bar in D = 5 μm (D-F). Bar in G = 10 μm. Bar in inset= 5 μm.

In view of the TnI requirements for cell proliferation, we questioned whether this protein will affect tumor proliferation. Consistent with the strategy followed in the TnI excess-of-function study, we tested overgrowths originated by *lgl^-^/Ras^V12^*and *N^intra^*. In the first case, mosaics yield a large hyperplasia, but the joint depletion of TnI greatly prevents it (Figure 4A-4C). Also, in wing *lgl↓/Ras^V12^* clones, the mutant cells are located at both apical and basal zones of the epithelium (see Z-axis view in Figure 4A). However, the simultaneous down-regulation of TnI induces basal positioning and extrusion (Figure 4B Z axis view). The cell extrusion process includes the recruitment of Nimrod-positive hemocytes as in other cell competition events [21] (Figure 4D-4F). These hemocytes engulf the TnI^RNAi^ expressing cells prior to cell extrusion (Figure 4G). Larvae carrying *lgl↓/Ras^V12^* clones fail to reach adulthood but the simultaneous down-regulation of TnI allows about 20% adult survival (Figure S5A). The survivors, however, live shorter than 7 days possibly because of the deleterious effects of multiple clones in various organs induced by the *act-Gal4* cassette (see M&M). Interestingly, routine inspection of larvae with *lgl↓/Ras^V12^* clones revealed 5-10% of cases with one or several dark inclusion bodies incrusted in the fat body (Figure 4H). Upon dissection, these bodies are constituted by GFP marked materials, hence derived from mutant cells (Figure 4I). When TnI is co-down regulated, inclusion bodies are about four times larger than those found in *lgl↓/Ras^V12^* larvae (Figure 4J) and are about three times more frequent. As it seems, the TnI-dependent cell elimination overrates the *lgl↓/Ras^V12^*growth. These features suggest that inclusion bodies are neoplasias of melanotic tumor cells following their extrusion from the original tissues. Presumably, these encapsulated extrusions will allow a higher survival rate of adults (Figure S5A). The issue, however, was not investigated further at this point. Eye mosaics also exhibited suppression of *lgl↓/Ras^V12^* overgrowths by TnI (Figure S5B, C). Following the same trend, the severe decrease of adult viability caused by *N^intra^*, expressed under *gmr-Gal4* driver, was largely suppressed by the simultaneous depletion of TnI (Figure 4K).

### Targeting mammalian TNNI severely hinders tumor growth

**Figure 5:**
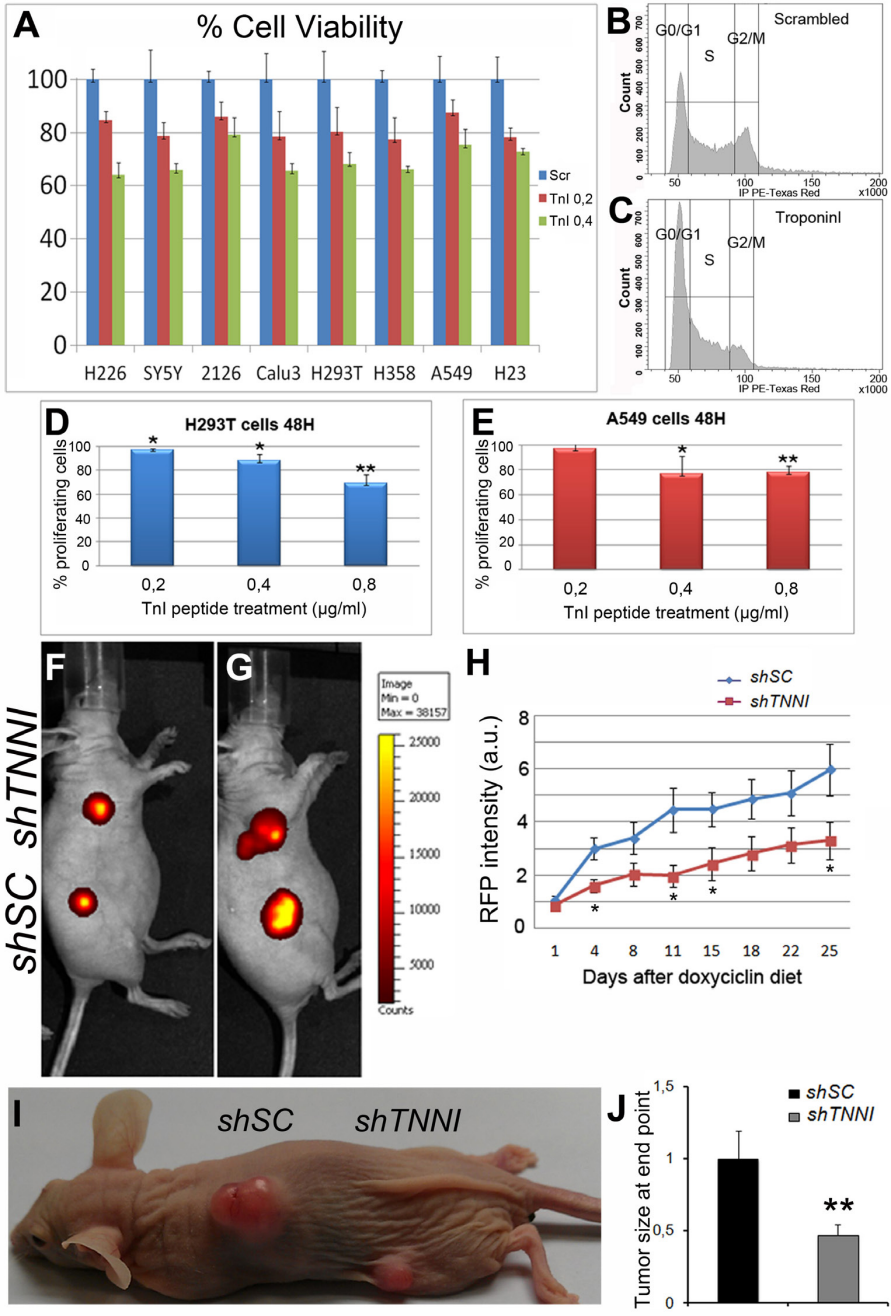
Human tumor growth can be reduced by *TNNI1* down regulation **A.** Cultured cell lines, normal and tumorigenic, are slowed down by treatment with a human TnI peptide in a dosage-dependent manner. H293T=fibroblasts used as normal control, SY5Y = neuroblastoma, H226 = lung adenocarcinoma, H358 = lung adenocarcinoma, A549 = lung adenocarcinoma, Calu3 = lung adenocarcinoma, 2126 = lung adenocarcinoma, H23 = lung adenocarcinoma. Blue = Treatment with scrambled peptide. Red = 0.2 μg/ml. Green = 0.4 μg/ml. **B.**, **C.** Cultured cells incubated with the TnI peptide arrest in G_0_/G_1_ mostly. **D.** Percentage of H293T proliferating cells after 48 h of TnI peptide treatment at various concentrations relative to scrambled treatment (0,8μg/ml). **E.** Equivalent experiments with A549 cells. Experiments were done in triplicates. Note the dosage effect in the reduction of proliferation. **F.**, **G.** IVIS images of the same mouse at day 1^st^(F) and day 25^th^(G) after doxycycline diet initiation. Note that cell density (color coded) of *shTNNI1*-expressing tumor is substantially lower than the control *shSC*-expressing tumor. **H.** Time course of tumor growth quantified by RFP signal acquisition during the longitudinal study. Five mice with two tumors each were used. A total of 10 tumors *shSC* and 10 tumors *shTNNI1* per time point were measured. **I.** Tumor appearance at experiment end point (day 25^th^). **J.** Quantification of tumor size normalized to control *shSC* tumors.

The *in vivo* effects of TnI on *Drosophila* hyper- and neoplasias invited to assay the equivalent effects on human cells. Out of the three TNNI encoding genes known in humans, we focused on the slow TNNI, *TNNI1*, because it shows the highest increase of expression levels in human tumors (Figure 1). In addition, *TNNI1* is the closest sequence homologue to the single *Drosophila* TnI gene. Seven different human tumor cell lines of various oncogenic origins [40-41] and the non-oncogenic embryonic kidney cell line (HEK293T) as control [42] were treated with a peptide corresponding to the 93-116 residues of human TNNI1. This peptide corresponds to the active center of the protein and, in effect, should recognize TNNI1 substrates and act as a dominant negative form. The data show a significant (30-40%) proliferation reduction in a dose dependent manner with respect to tumor cell lines treated with a scrambled peptide as control (Figure 5A) (see Suppl. Information for peptides sequences). After characterization in a flow-cytometer, treated cells arrested mainly in G_0_/G_1_ (Figure 5B, 5C) and the fraction of proliferating cells 48h after treatment was inversely proportional to the peptide concentration used (Figure 5D, 5E).

Since the anti-TnI treatment seemed to have a direct effect on tumor cells in culture, we extended the analysis to an organism setting. For this purpose, we used the human non-small cell lung carcinoma line A549, which expresses high levels of TNNI1 (Figure S6A, B), and a shRNA construct that allows expression concomitant with red fluorescent protein (RFP) upon induction by doxycycline feeding. We generated stable cell lines with *shTNNI1* or scrambled control *shSC*. First, we validated the shRNAs as down-regulators of TNNI1 expression following doxycycline administration (2μg/ml) (Figure S6C, D). Next, we injected both A549 stable cell lines in nude mice, and tumors were allowed to grow for one week in the absence of doxycycline. Mice were then treated with doxycycline and their growth rate was measured by *in vivo* imaging of RFP signal. The growth rate of *shTNNI1*/A549 tumors was significantly slower than *shSC*/A549 (Figure 5F-5H). Also, the tumor size at the experiment end point confirms that the size of *shTNNI1*/A549 tumors was clearly diminished when compared to *shSC*/A549 tumors (Figure 5I, 5J). Taking together, this set of experiments demonstrates that the pro-tumor role of Troponin-I described in flies is conserved in human cells and, thus, it may become of potential therapeutic interest.

## DISCUSSION

We show here that the excess of TnI stimulates proliferation and, if concomitant with certain oncogenic signals, tumors can be formed. The mechanism by which TnI increases cell proliferation includes changes in transcription of selected genes some of which have been identified here. By contrast, TnI depletion renders the cells unfit and subject to competition by neighbors and, if simultaneous with certain oncogenic signals, tumor growth can be largely restrained in fly and human cells. Thus, although considered hereto as a muscle specific protein, TnI is now revealed as a player of cell biology with functions in the nucleus and in the cytoplasm of most, if not all, cell types. Being an actin-binding protein, TnI should play all these functions in relation to that partner.

Based on the high content of actin within the nucleus, it has been proposed that a force-generating motor could help to move the RNA polymerase complex [43]. In this context, it is plausible that TnI could regulate this actin-based motor. Nevertheless, since TnI does not affect transcription of all genes assayed here, it seems likely that the transcriptional role of TnI would be gene-specific or physiological cell status-specific. It is worth noting that *Myc, Ras* or *Notch* transcription is not altered. Thus, akin to these classical oncogenes, TnI exhibits specificity in the targeted genes. This is in line with, for example, the fact that the transcriptional targets of *Ras* are different from those of *Notch* [44]. The specificities of these targeted gene repertoires would sustain the synergy when they coincide in the same cell. From a conceptual point of view, oncogenesis may be viewed as a convergence of several mis-regulated processes that lead a cell towards unrestrained proliferation. If more than two of these mechanisms converge (e.g.: Lgl deficit, Ras^V12^, TnI excess), their synergy may yield massive overgrowths.

The novel transcriptional activity of fly TnI may explain the high prevalence of *TNNI1*over-expression in certain human cancers as reported in the databases. Not all types of human cancer, however, show correlation with high *TNNI1* expression (e.g.: squamous lung cancer). These cases are consistent with the specificity of the transcriptional targets of TnI vs that of other oncogenes. Thus, TnI over-expression is not a consequence of mutations in oncogenes, as much as the over-expression of TnI does not elicit transcriptional changes in any of the tested oncogenes.

TnI depletion reduces proliferation and causes some form of unfit state with cell deleterious effects that may be followed by cell extrusion (wing disc). One of the proposed mechanisms for wing cells extrusion is dependent on actin/myosin, [45-46]; which justifies the involvement of TnI. Since TnI, and its partner actin, can localize in the nucleus as well as in the cytoplasm, it follows that changes in TnI levels will affect a variety of cellular processes including gene transcription and cytoplasmic signaling through actin cytoskeleton. Finally, the TNNI1 peptide and the TNNI1 shRNAi used here severely limited tumor growth. It should be noted that the human cell lines chosen in this study are notoriously aggressive and resilient to anti-tumor agents [41]. The proven effectiveness of blocking Troponin I to restrain a diversity of tumor cell types may open the possibility to further consider this protein as a new anti-tumor agent in cancers where TNNI1 is overexpressed.

## MATERIALS AND METHODS

### Fly strains and genetic crosses

The fly stocks used are from the Bloomington Stock Center (Fly Base) except as indicated. The TnI excess-of-function was elicited by the *PBac(WH)f06492* from Exelixis referred here as *UAS-TnI^f06492^*. This transposon is inserted 5′ of *wupA*, and the full sequence information can be obtained from Fly Base. As null TnI mutations we used two rearrangements located the regulatory region of the *wupA* gene, *Df(1) TnI^23437^* and *In(1)PL87* [39]. *Dp(1;3)JC153* covers *wupA* [47]. See Suppl. Information.

### Mosaic generation and clone size measurement

FRT-mitotic recombination clones were obtained by delivering a heat shock (1hour at 37°C) during 2^nd^ instar larvae. 48 or 72 hours after heat shock 3^rd^ instar wandering larvae were dissected. FLP-out clones were obtained through the same protocol but with different heat shock conditions (8 minutes at 37°C). Control cultures were run in parallel. Wandering LIII larvae were dissected for clone screening. A software-assisted area measurement (Bitplane's Imaris Surface) was used to obtain clone area and cell size. Cell profiles are identified by the myrRFP reporter and cell nuclei are revealed by DAPI. See Suppl. Information.

### Western blotting

Western blots were obtained by standard procedures (Invitrogen) and protein bands were quantified by densitometry using ImageJ software with the “GelAnalyzer” option (See further details in http://www.di.uq.edu.au/sparqimagejblots).

### Immunohistochemistry

Tissue samples were fixed in formaldehyde 4% 25 min and stained according to standard protocols. The J4 polyclonal antibody identifies the nuclear TnI as described previously (Sahota el at., 2009). The SC1 mouse monoclonal antibody was raised against the peptide PDGDPSKFAS (Abmart Inc.). Primary antibodies: anti-Active caspase3 (1/100 Cell Signaling), anti-Nimrod (1/100 a gift from I. Andó), anti-Tub (1/100 SIGMA) and anti-β-galactosidase (1/50 DSHB). Fluorescent secondary antibodies Alexa 488, 569 and 647 were used (1/200 Invitrogen). All images were obtained with a LEICA TCS-SP5 confocal microscope.

### Quantitative qRT-PCR and primer's sequences

For qRT-PCR assays, RNA was extracted with Trizol (Invitrogen) according to standard procedures. To prevent genomic DNA contamination all RNA samples were treated with DNaseI according to manufacturer's procedures (30 min at 37°C). Primers were designed to anneal in different exons from each gene, later qPCR products were run in 2% agarose gels to analyze the bands. All the products correspond with their expected length, which indicates that no genomic DNA contamination was present. Assays were performed in triplicates using RNApolII as a housekeeping gene. *Drosophila* Troponin-I TaqMan Gene Expression probe (Applied Biosystems) was used. (See Suppl. Information for further details).

### Statistics

The area of GFP positive clones was quantified using Image-J A 1.44a software. Averages and standard deviations (SD) were calculated from the ratio GFP/DAPI area. Statistical significance was calculated with the two-tailed Student's *t*-test. Significance levels are indicated as *p < 0,05 ­**p < 0,005 ***p < 0,001. Number of samples *N* > 8 animals in all cases.

## SUPPLEMENTARY MATERIALS FIGURES



## References

[1] Vassylyev DG, Takeda S, Wakatsuki S, Maeda K, Maeda Y (1998). Crystal structure of troponin C in complex with troponin I fragment at 2.3-A resolution. Proc Natl Acad Sci U S A.

[2] Patchell VB, Gallon CE, Hodgkin MA, Fattoum A, Perry SV, Levine BA (2002). The inhibitory region of troponin-I alters the ability of F-actin to interact with different segments of myosin. Eur J Biochem.

[3] Takeda S, Yamashita A, Maeda K, Maeda Y (2003). Structure of the core domain of human cardiac troponin in the Ca(2+)-saturated form. Nature.

[4] Sahota VK, Grau BF, Mansilla A, Ferrus A (2009). Troponin I and Tropomyosin regulate chromosomal stability and cell polarity. J Cell Sci.

[5] Moses MA, Wiederschain D, Wu I, Fernandez CA, Ghazizadeh V, Lane WS, Flynn E, Sytkowski A, Tao T, Langer R (1999). Troponin I is present in human cartilage and inhibits angiogenesis. Proc Natl Acad Sci U S A.

[6] Kern BE, Balcom JH, Antoniu BA, Warshaw AL, Fernandez-del Castillo C (2003). Troponin I peptide (Glu94-Leu123), a cartilage-derived angiogenesis inhibitor: in vitro and in vivo effects on human endothelial cells and on pancreatic cancer. J Gastrointest Surg.

[7] Slaughter DP, Southwick HW, Smejkal W (1953). Field cancerization in oral stratified squamous epithelium; clinical implications of multicentric origin. Cancer.

[8] Casas-Tinto S, Torres M, Moreno E (2011). The flower code and cancer development. Clin Transl Oncol.

[9] Baker NE, Li W (2008). Cell competition and its possible relation to cancer. Cancer Res.

[10] de Beco S, Ziosi M, Johnston LA (2012). New frontiers in cell competition. Dev Dyn.

[11] Morata G, Ripoll P (1975). Minutes: mutants of drosophila autonomously affecting cell division rate. Dev Biol.

[12] Moreno E, Basler K (2004). dMyc transforms cells into super-competitors. Cell.

[13] Zoranovic T, Grmai L, Bach EA (2013). Regulation of proliferation, cell competition, and cellular growth by the Drosophila JAK-STAT pathway. JAKSTAT.

[14] Kolahgar G, Suijkerbuijk SJ, Kucinski I, Poirier EZ, Mansour S, Simons BD, Piddini E (2015). Cell Competition Modifies Adult Stem Cell and Tissue Population Dynamics in a JAK-STAT-Dependent Manner. Dev Cell.

[15] Rodrigues AB, Zoranovic T, Ayala-Camargo A, Grewal S, Reyes-Robles T, Krasny M, Wu DC, Johnston LA, Bach EA (2012). Activated STAT regulates growth and induces competitive interactions independently of Myc, Yorkie, Wingless and ribosome biogenesis. Development.

[16] de la Cova C, Abril M, Bellosta P, Gallant P, Johnston LA (2004). Drosophila myc regulates organ size by inducing cell competition. Cell.

[17] Claveria C, Giovinazzo G, Sierra R, Torres M (2013). Myc-driven endogenous cell competition in the early mammalian embryo. Nature.

[18] Mamada H, Sato T, Ota M, Sasaki H (2015). Cell competition in mouse NIH3T3 embryonic fibroblasts is controlled by the activity of Tead family proteins and Myc. J Cell Sci.

[19] Merino MM, Rhiner C, Portela M, Moreno E (2013). “Fitness fingerprints” mediate physiological culling of unwanted neurons in Drosophila. Curr Biol.

[20] Rhiner C, Lopez-Gay JM, Soldini D, Casas-Tinto S, Martin FA, Lombardia L, Moreno E (2010). Flower forms an extracellular code that reveals the fitness of a cell to its neighbors in Drosophila. Dev Cell.

[21] Lolo FN, Casas-Tinto S, Moreno E (2012). Cell competition time line: winners kill losers, which are extruded and engulfed by hemocytes. Cell Rep.

[22] Gyorffy B, Surowiak P, Budczies J, Lanczky A (2013). Online survival analysis software to assess the prognostic value of biomarkers using transcriptomic data in non-small-cell lung cancer. PLoS One.

[23] Tamori Y, Bialucha CU, Tian AG, Kajita M, Huang YC, Norman M, Harrison N, Poulton J, Ivanovitch K, Disch L, Liu T, Deng WM, Fujita Y (2010). Involvement of Lgl and Mahjong/VprBP in cell competition. PLoS Biol.

[24] Grzeschik NA, Amin N, Secombe J, Brumby AM, Richardson HE (2007). Abnormalities in cell proliferation and apico-basal cell polarity are separable in Drosophila lgl mutant clones in the developing eye. Dev Biol.

[25] Froldi F, Ziosi M, Garoia F, Pession A, Grzeschik NA, Bellosta P, Strand D, Richardson HE, Grifoni D (2010). The lethal giant larvae tumour suppressor mutation requires dMyc oncoprotein to promote clonal malignancy. BMC Biol.

[26] Menendez J, Perez-Garijo A, Calleja M, Morata G (2010). A tumor-suppressing mechanism in Drosophila involving cell competition and the Hippo pathway. Proc Natl Acad Sci U S A.

[27] Zuber J, Tchernitsa OI, Hinzmann B, Schmitz AC, Grips M, Hellriegel M, Sers C, Rosenthal A, Schafer R (2000). A genome-wide survey of RAS transformation targets. Nat Genet.

[28] Gyorffy B, Schafer R (2010). Biomarkers downstream of RAS: a search for robust transcriptional targets. Curr Cancer Drug Targets.

[29] Brogiolo W, Stocker H, Ikeya T, Rintelen F, Fernandez R, Hafen E (2001). An evolutionarily conserved function of the Drosophila insulin receptor and insulin-like peptides in growth control. Curr Biol.

[30] Guvakova MA, Lee WS, Furstenau DK, Prabakaran I, Li DC, Hung R, Kushnir N (2014). The small GTPase Rap1 promotes cell movement rather than stabilizes adhesion in epithelial cells responding to insulin-like growth factor I. Biochem J.

[31] Garelli A, Gontijo AM, Miguela V, Caparros E, Dominguez M (2012). Imaginal discs secrete insulin-like peptide 8 to mediate plasticity of growth and maturation. Science.

[32] Colombani J, Andersen DS, Boulan L, Boone E, Romero N, Virolle V, Texada M, Leopold P (2015). Drosophila Lgr3 Couples Organ Growth with Maturation and Ensures Developmental Stability. Curr Biol.

[33] Hariharan IK (2005). Ras and Rap: are former enemies now friends?. Dev Cell.

[34] Mishra S, Smolik SM, Forte MA, Stork PJ (2005). Ras-independent activation of ERK signaling via the torso receptor tyrosine kinase is mediated by Rap1. Curr Biol.

[35] Rewitz KF, Yamanaka N, Gilbert LI, O'Connor MB (2009). The insect neuropeptide PTTH activates receptor tyrosine kinase torso to initiate metamorphosis. Science.

[36] Portela M, Casas-Tinto S, Rhiner C, Lopez-Gay JM, Dominguez O, Soldini D, Moreno E (2010). Drosophila SPARC is a self-protective signal expressed by loser cells during cell competition. Dev Cell.

[37] Moreno E, Basler K, Morata G (2002). Cells compete for decapentaplegic survival factor to prevent apoptosis in Drosophila wing development. Nature.

[38] Wang SL, Hawkins CJ, Yoo SJ, Muller HA, Hay BA (1999). The Drosophila caspase inhibitor DIAP1 is essential for cell survival and is negatively regulated by HID. Cell.

[39] Marin MC, Rodriguez JR, Ferrus A (2004). Transcription of Drosophila troponin I gene is regulated by two conserved, functionally identical, synergistic elements. Mol Biol Cell.

[40] Biedler JL, Roffler-Tarlov S, Schachner M, Freedman LS (1978). Multiple neurotransmitter synthesis by human neuroblastoma cell lines and clones. Cancer Res.

[41] Blanco R, Iwakawa R, Tang M, Kohno T, Angulo B, Pio R, Montuenga LM, Minna JD, Yokota J, Sanchez-Cespedes M (2009). A gene-alteration profile of human lung cancer cell lines. Hum Mutat.

[42] Graham FL, Smiley J, Russell WC, Nairn R (1977). Characteristics of a human cell line transformed by DNA from human adenovirus type 5. J Gen Virol.

[43] Ye J, Zhao J, Hoffmann-Rohrer U, Grummt I (2008). Nuclear myosin I acts in concert with polymeric actin to drive RNA polymerase I transcription. Genes Dev.

[44] Sundaram MV (2005). The love-hate relationship between Ras and Notch. Genes Dev.

[45] Rosenblatt J, Raff MC, Cramer LP (2001). An epithelial cell destined for apoptosis signals its neighbors to extrude it by an actin- and myosin-dependent mechanism. Curr Biol.

[46] Slattum G, McGee KM, Rosenblatt J (2009). P115 RhoGEF and microtubules decide the direction apoptotic cells extrude from an epithelium. J Cell Biol.

[47] Ferrus A, Llamazares S, de la Pompa JL, Tanouye MA, Pongs O (1990). Genetic analysis of the Shaker gene complex of Drosophila melanogaster. Genetics.

