# Supplementary material for: Troponin-I enhances and is required for oncogenic overgrowth
Source: Oncotarget. 2016 Jul 15;7(33):52631–42. doi: 10.18632/oncotarget.10616 (PMC5288137; doi:10.18632/oncotarget.10616)
Supplement: Supplementary file 1 [file oncotarget-07-52631-s001.pdf]

Troponin-I enhances and is required for oncogenic overgrowth

Supplementary Material

EXPERIMENTAL PROCEDURES

Fly strains and genetic crosses. The effectiveness of the *PBac(WH)f06492* from Exelixis construct to over-express TnI was validated by qRT-PCR assay in *tub-Gal4LL7>UAS-TnI*^{f06492} adults. Among the other available inserts in *wupA*, *P(XP)d07274* cannot be used due to its bidirectional configuration and *P(EP)G20589* is not adequate for the over-expression experiments because it is a mutant allele for *wupA* based on the lack of complementation with *hdp* and *TnI*^{23437} alleles. To induce TnI loss-of-function cell mosaics, we tested three RNAi lines: *P{TRiP.JF02172}attP2* (BL#31893), *P[GD10595]v34196* (from VDRC) and L9 (generated in our lab and directed against an embryonic form of TnI). Only the first proved effective against *wupA* expression and to yield wings up phenotype when driven to muscles by*24B-Gal4*. Thus, we used it throughout referred as *UAS-TnI*^{RNAi}. In experiments with the *rotund* (*rn*) domains, females of genotype *elavGal80 ; rnGal4, UAS-GFP*^{nls}*/TM6* were crossed to *UAS-TnI*^{RNAi} (BL#31893) males. The *elavGal80* repressor was used to prevent TnI knockdown in the nervous system. The *lgl*^{RNAi} (GD51249) and *fwe*^{RNAi} (KK104993) lines were from the VDRC repository. Eye experiments were performed with *gmr-Gal4 [1]* while construct *UAS-Sparc* was from E. Moreno. Lines *UAS-bsk*^{DN} (BL#6409) and *UAS-Dpp-GFP-H2* were from our fly collection. FLP-out mosaics were obtained by heat shock of larvae carrying *hs-FLP*; *actin-FRT-yellow*$^{+}$*-Stop-FRT-Gal4,UAS-GFP/+* combined with the desired *UAS-x* construct. Two *act-FRT-Gal4* cassettes, inserted in chromosome II or III, were used according to the desired expression strength.

Mosaic generation and clone size measurement. Crosses were set with 10 females and 10 males per vial at 25ºC and vials were changed every 72 hours to avoid overcrowding. A software-assisted area measurement (Bitplane's Imaris Surface) was used to obtain clone area and cell size. Briefly, files from the confocal microscope were loaded in this software as a 3D image. The "surface" option was chosen to measure the areas occupied by DAPI or GFP/RFP pixels from the entire 3D image. The area of the clone (GFP or RFP in each experiment) was calculated and divided by DAPI area. As a result, the percentage of wing imaginal disc occupied by genetically marked cells was

represented. The quotient between GFP/DAPI indicates the total clonal area in the disc. Cell profiles were labelled by the myristoylated form of RFP and the data were treated with a similar protocol for calculations of total clone area. Caspase 3 quantification was performed with the “surface” option of the same software. To measure activated C3 signals in the anterior versus posterior wing compartments, a green mask to occlude one compartment was applied. The ratio of posterior versus anterior C3 positive area was represented.

Quantitative qRT-PCR and primer sequences. For qRT-PCR assays, adults were obtained from crosses indicated in the corresponding figure legends. Crosses were maintained at 17ºC during development and, after eclosion, 5 days old adult flies were shifted to 29ºC for 36 hours, then groups of 30 males were frozen in liquid nitrogen. RNA was extracted with Trizol (Invitrogen) according to standard procedures. To prevent genomic DNA contamination all RNA samples were treated with DNaseI according to manufacturer´s procedures (30 min at 37ºC). Primers were designed to anneal in different exons from each gene, later qPCR products were run in 2% agarose gels to analyze the bands. All the products correspond with their expected length, which indicates that no genomic DNA contamination was present. Assays were performed in triplicates using RNApolII as a housekeeping gene. *Drosophila* Troponin-I TaqMan Gene Expression probe (Applied Biosystems) was used.

The following forward (F) and reversal (R) primers were used to monitor the genes assayed in the qRT-PCR assays:

CDK2 F-5´GTCATTTCCGGCAACAATCTGT 3´

CDK2 R- 5´CATCAGATATTAGATGCCGTCGG 3´

Rap1 F-5´ AAGAACCTGGCCACCCAGTT 3´

Rap1 R-5´ ACAAGAAGTCGCCCGAGAAG 3´

InR F-5´ GGAGCGAATCAAACCCAACA 3´

InR R-5´ CGATTTGATAAACGACGCCA 3´

dILP8 F-5´GATGAAGAAGTTCGCGATGGA 3´

dILP8 R-5´AAGCAGCTATCCAATGGGTGGCTACCTGAA 3´

Notch F-5´ CGAAATTGCGGTACCGAATG 3´

Notch R-5´ GTGAACGATGCGAGACGAAG 3´

dMyc F-5´ TGGAGACCCCCTCAGATTCC 3´

dMyc R-5´TCACATGAAGCAAAAACCGC 3´

Ras F-5´GATTCATCGCAGGGCAACTC 3´

Ras R-5´CCAAAAATCTCATTGATGCCG 3´

ptc F-5´CAGCTGTTGGGTCCGGAAT 3´

ptc R-5´ ACTTCGAGACCGTGGAGCAGT 3´

Rab5 F-5´ CGATAGGTGCGGCCTTTCT 3´

Rab5 R-5´ TATTATCGAGGAGCGCAGGC 3´

Stat92 F-5´ GGCGGCTTACTACGAGGAGAA 3´

Stat92 R-5´ AAAGCTTCTATCCACGCGCA 3´

Dad F-5´CTCACACCAGCAGGCAATCA 3´

Dad R-5´ CGATTCACCCACCCTGTCC 3´

dIAP1 F-5´ GCCGGCGACAAAGTTAAATG 3´

dIAP1 R-5´ GCATTAGATCGCATCCTGCC 3´

ex F-5´ ATCACCAGGCTCGCGATTC 3´

ex R-5´CATGTCTGCACTTTGCCACG 3´

CycA F-5´GGCCTCCCTAGTCGACAAA 3´

CycA R-5´TATCAGCGTGGGCACTGAAA 3´

Cell cycle analysis and anti TNNI1 peptides. Cells were permeabilized using PBS with 0.1% Triton X-100 (Sigma) and incubated 30 min with 100 μg/ml Ribonuclease A (Sigma) and 50 μg/ml Propidium Iodide (PI) (Sigma). Argon laser excitation at 488 nm was used to measure PI fluorescence through a band-pass 616/23 nm filter using a FACSAria flow cytometer (BD Biosciences, San Diego, CA). Debris and duplets were excluded from the analysis and a minimum of 10^4 cells were acquired per experiment. FACSDiva software (BD Biosciences) was utilized for cell cycle stages definition. To generate stable cell lines, human non-small cell lung cancer cells A549 were infected with the supernatant of 293 cells transfected with pΔ8.9 and pVSVG plus the combination of human TRPZ lentiviral shRNAmir target gene set RHS4740-NM_003281 clones V3THS_405472 and V3THS_339233, or with the TRPZ scramble

(shSC) from Thermo Scientific Open Biosystems following manufacturer instructions. After puromycin selection, cells were induced 24 hours with 2 µg/ml of doxycycline (Sigma-Aldrich) to induce expression of shRNAmir and RFP. Cells were RFP sorted using a FACS Aria cell sorter (BD Biosciences) and amplified in the absence of doxycycline. To analyze the level of down-regulation, sorted cells were induced with doxycycline for 48h and analyzed for TNNI protein expression by Western blot. For peptide treatments, we applied the water soluble EIKDLKLEVMDLRGKFKRPPLRRV (anti-TNNI1) or DEGKVPDFLRKVKIKELRMRLRLP (scrambled control) at the desired concentrations to the culture media. Both peptides were synthesized at the proteomic facility of the CIB-CSIC.

Mice. Mice were housed at the serum pathogen-free (SPF) barrier area of the Spanish National Cancer Research Center (CNIO), Madrid. Mice were observed daily and sacrificed when they showed overt signs of morbidity or tumours, in accordance to the *Guidelines for Humane Endpoints for Animals Used in Biomedical Research*. $2.5X10^6$ A549 cells infected with *shTNNI1* or *shSC*, were injected subcutaneously at both flanks of nude mice (*FoxN1*$^{-/-}$, Harlan) as described [2]. 7 days after injection, mice were fed with doxycycline (625mg/Kg; Harlan) to induce shRNAmir and RFP expression. Tumour size was followed by fluorescence as described [3] using DsRed filters (500-550nm and 575-650nm) and measured twice per week using an IVIS-200 small animal imaging system (Caliper Life Sciences). Photons emitted (total flux expressed as photons per second and corrected by F-Stop) from tumoral masses were quantified using Living Image software (Caliper Life Sciences).

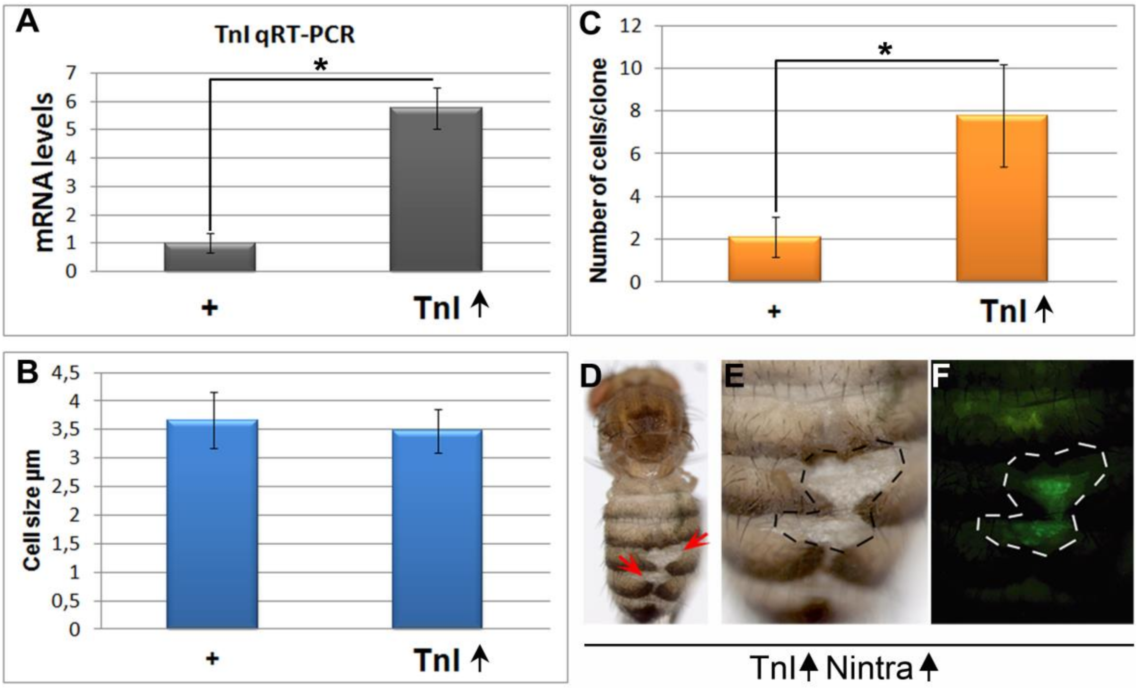

Supplementary Figure S1.- TnI over-expression increases proliferation. A) Quantitative RT-PCR assays with whole adults demonstrate that the *P(w^{+})^{WH}UAS-TnI^{f06492}* construct effectively increases TnI expression. Genotypes: *tub-Gal4^{LL7}/UAS-LacZ* (control, +) and *tub-Gal4^{LL7}/UAS-TnI^{f06492}* (TnI↑). **B,C**) The quantification of TnI over-expression does not change cell size (**B**) but it increases four times the number of cells per clone (**C**) at 48 h AHS. Data correspond to main **Fig. 1A,B**. Genotype: *UAS-TnI^{f06492}/hs-FLP ; +/+; actin-FRT-Stop-FRT-Gal4,UAS-myrRFP*/+. Note that cell profiles are visualized by the myristoylated form of RFP, *myrRFP,* enabling to evaluate cell size in single confocal planes. Data quantification was obtained through the Imaris Bitplane software which handles 3D images. **D-F**) Exceptional adult survivors from larvae with multiple clones co-expressing *UAS-TnI^{f06492}* and *N^{intra}* show abnormal outgrowths (arrows in **D**) marked with GFP (**F**), which demonstrates their clonal origin. Dashed line in **E** indicates the externally visible outgrowth in **D** but additional GFP marked cells can be seen which are underneath the cuticle (**F**). Genotype: *UAS-TnI^{f06492}/hs-FLP*; *UAS-N^{intra}/actin-FRT-Stop-FRT-Gal4,UAS-GFP*.

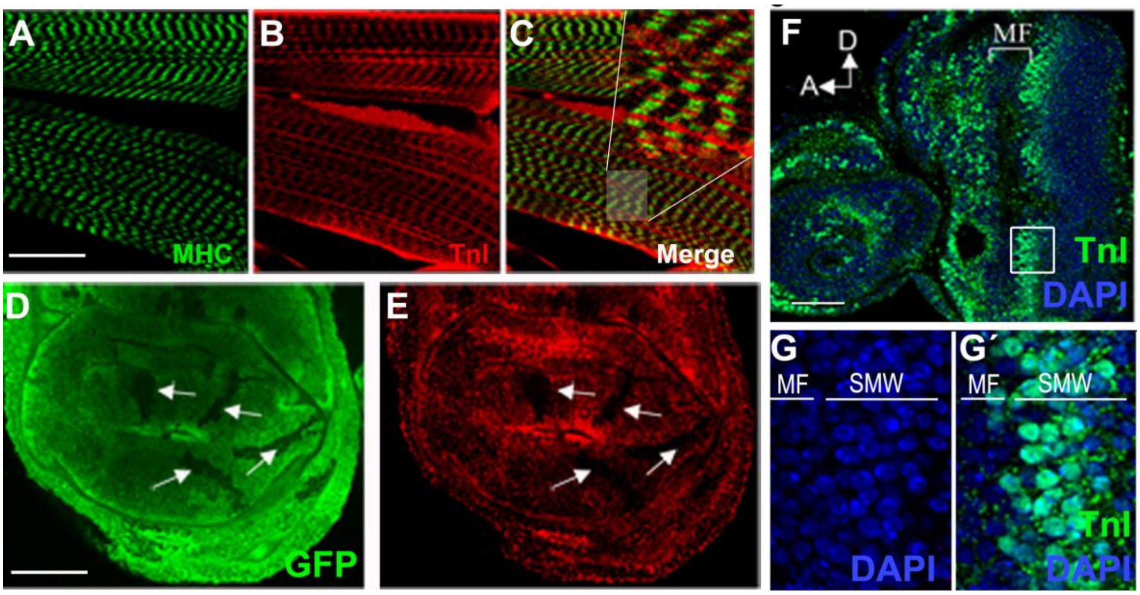

Supplementary Figure S2.- Antibody validation and subcellular localization of TnI. A-C) Adult indirect flight muscles stained with monoclonal antibody SC1 (red). The signal identifies actin based thin filaments which is the canonical location of Troponin I in muscles (see inset in **C**). Myosin is visualized by a Mhc-GFP construct. Genotype: *Canton-S*. **D,E**) Validation of the SC1 antibody (red) in wing disc FLP-out clones for a TnI null mutation that deletes 2Kb of the regulatory region of the gene (genotype: ♀f^5*Df(1)TnI*^{23437} *FRT18A / y,M(1)O*^{Sp}*,UbiGFP,FRT18A; hs-FLP38/+*). Note that the negative immunosignal (arrows) coincides with the mutant clones. The relatively large size of the TnI null clones is due to their Minute$^+$ condition on a Minute background. **F**) Eye disc stained with J4 antibody shows nuclear TnI in relation to the morphogenetic furrow (MF). A=Anterior, D= Dorsal. **G, G'**) Detail of the disc in **F** (inset). Note the nuclear location of TnI in cells immediately posterior to the furrow (second mitotic wave, SMW) but lack of expression in the rest of the posterior disc were mitotic activity has ceased. Images in **A, B, C, D** and **E** are single confocal planes. Images in **F** and **G** are maximal projections. Bar in **A** = 10 µm (**A-C**). Bar in **D** = 50µm (**D, E**). Bar in **G** = 15 µm.

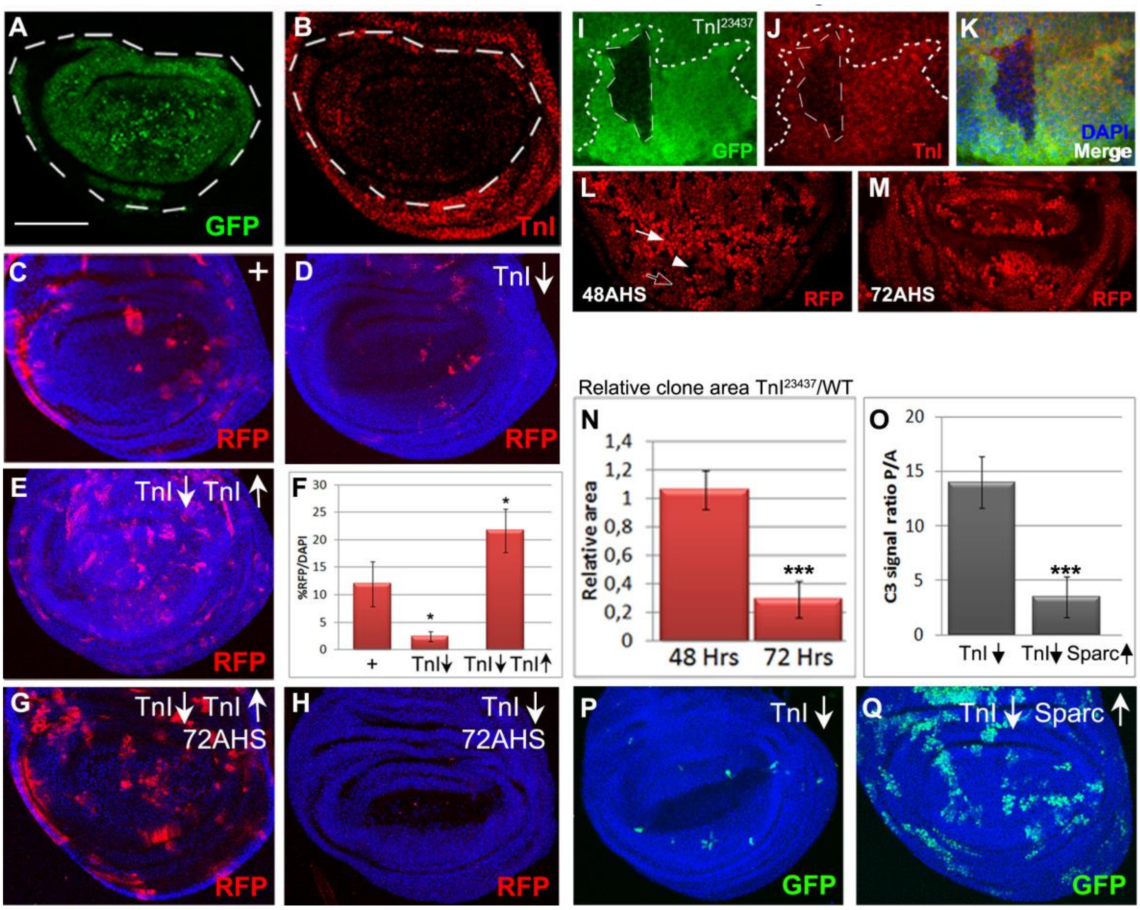

Supplementary Figure S3.- RNAi validation and time course expression of TnI. **A,B**) Validation of the TnI^{RNAi} in the wing pouch domain (*rotund-Gal4*) stained with J4 antibody. Note the severe reduction of immune signal in the wing pouch (dashed line) with respect to the rest of the wing disc. **C-F)** The size of 48h FLP-out clones in a normal wing disc (**C**), is greatly reduced when cells express TnI^{RNAi} (**D**). However, the effect is counterbalanced if the *UAS-TnI* construct is co-expressed (**E**). Data are quantified in **F**. Genotypes: *+/hs-FLP*; *UAS-LacZ/actin-FRT-Stop-FRT-Gal4,UAS-RFP* (**C**), *hs-FLP/+* ; *UAS-LacZ/actin-FRT-Stop-FRT-Gal4,UAS-RFP;* *UAS-*TnI^{RNAi}*/+* (**D**) and *UAS-*TnI^{f06492}*/hs-FLP* ; *actin-FRT-Stop-FRT-Gal4,UAS-RFP/+* ; *+/UAS-*TnI^{RNAi} (**E**). **G,H**) The counterbalance is also evident at 72 h after clone induction. **I-K**) Twin clone configuration (genotype: ♀ f^5*Df(1)*TnI^{23437} *FRT18A/Ubi-GFP FRT18A; hs-FLP38/+*). Note that the TnI^{23437}/TnI^{23437} clone (dashed line) is much smaller than its strong GFP

marked twin (dotted line) which indicates proliferation defects in the TnI null cells. The TnI null cells still maintain a weak immune-TnI signal (**J**, red) illustrating the perdurance of the TnI protein. TnI is revealed by SC1 antibody. **L-N**) Comparison of 48 h AHS clones (**L**) versus 72 h AHS (**M**) TnI deficient clones. Note the three levels of RFP (red) signal; the strongest (arrow) corresponds to the wild type homozygous cells generated in twin with the homozygous TnI null cells which lack RFP signal (arrow head). The intermediate RFP intensity signal corresponds to the heterozygous neighboring cells. Genotype: ♀ *Ubi-mRFP*^{nls}*, hsFLP, FRT19A / f*^{5}*Df(1)TnI*^{23437} *FRT19A.* Data quantification is shown in (**N**). Note that data are not normalized, but expressed as the ratio between the TnI null (no RFP pixels in **L, M**) and the wildtype (strong red pixels in **L, M**) areas at 48 and 72 h AHS. **O-Q**) Data quantification of TnI-depleted FLP-out clones induced at 48h AHS, but rescued by the over-expression of *Sparc* (**O**), and representative images of these wing disc mosaics (**P, Q**). Genotypes: *+/hs-FLP*; *UAS-LacZ*/+; *actin-FRT-Stop-FRT-Gal4,UAS-GFP/UAS-TnI*^{RNAi} (**P**) and *+/hs-FLP*; *UAS-Sparc*/*actin-FRT-Stop-FRT-Gal4,UAS-GFP; UAS-TnI*^{RNAi}*/+* **(Q)**. Images are stacks of several confocal planes. Bar in **A** = 50 µm.

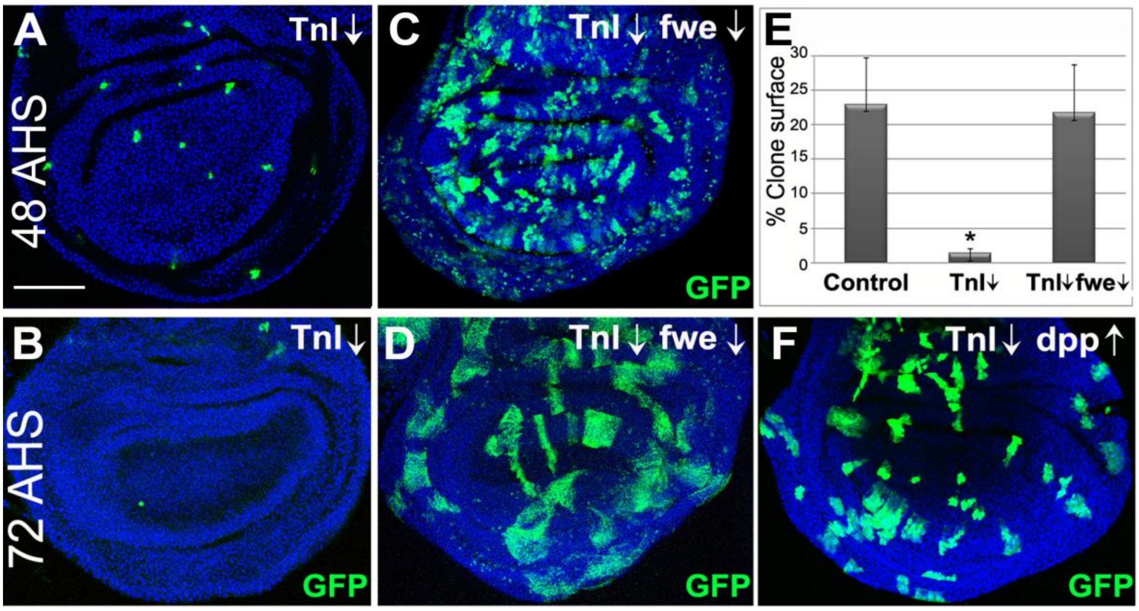

Supplementary Figure S4.- TnI is required for cell proliferation. A) FLP-out wing disc mosaics of TnI^{RNAi} expressing cells (GFP) do not reach the 4 cell stage by 48h after clone induction (AHS) (genotype: *+/hs-FLP*; *actin-FRT-Stop-FRT-Gal4,UAS-GFP/+ ; UAS-TnI^{RNAi}*/+). **B)** At 72 h post clone induction, most TnI deficient cells have been eliminated. **C,D)** The simultaneous down-regulation of *Flower* (*fwe*) allows further growth of TnI deficient clones at both time points. Genotype: *+/hs-FLP*; *actin-FRT-Stop-FRT-Gal4,UAS-GFP/UAS-fwe^{RNAi}; UAS-TnI^{RNAi}*/+). **E)** Quantifications of data from experiments shown in **A** and **C**, using as control the data from genotype *x/hs-FLP*; *actin-FRT-Stop-FRT-Gal4,UAS-GFP/UAS-LacZ*. In this way, the potential titration effect of UAS constructs is accounted for. **F)** Rescue of 72 h AHS clones by Dpp over-expression. Cell nuclei are marked by DAPI (blue). Genotype: *+/hs-FLP*; *UAS-dpp-GFP*/*actin-FRT-Stop-FRT-Gal4,UAS-GFP; UAS-TnI^{RNAi}*/+. Images are maximal projections. Bar in **A** = 50 µm in **A-F**.

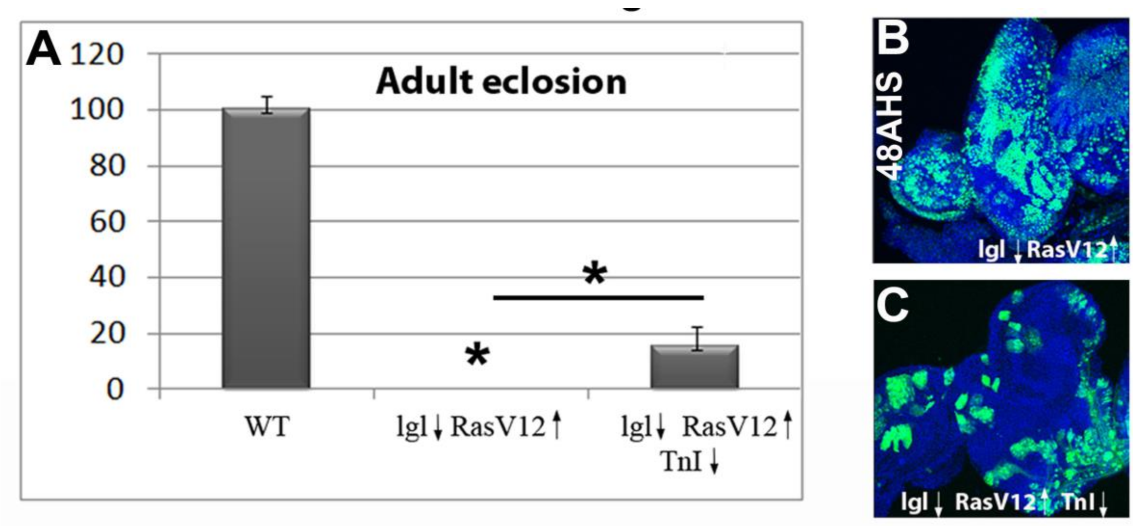

Supplementary Figure S5.- Rescue of oncogenic overgrowths by TnI depletion. A) Larvae with multiple FLP-out clones of *lgl↓/Ras*^{V12}cells do not yield viable adults unless TnI is co-down-regulated (20% viability). These adults, however, live shorter than seven days (see main text). **B,C**) In eye discs, TnI also shows repression of the *lgl↓/Ras*^{V12} overgrowths. Genotypes: ♀ *+/hs-FLP*; *actin-FRT-Stop-FRT-Gal4,UAS-GFP/UAS-RasV12; UAS-lgl*^{RNAi}*/+* (**B**) and ♀ *+/hs-FLP*; *actin-FRT-Stop-FRT-Gal4,UAS-GFP/UAS-RasV12; UAS-lgl*^{RNAi}*/UAS-TnI*^{RNAi} (**C**).

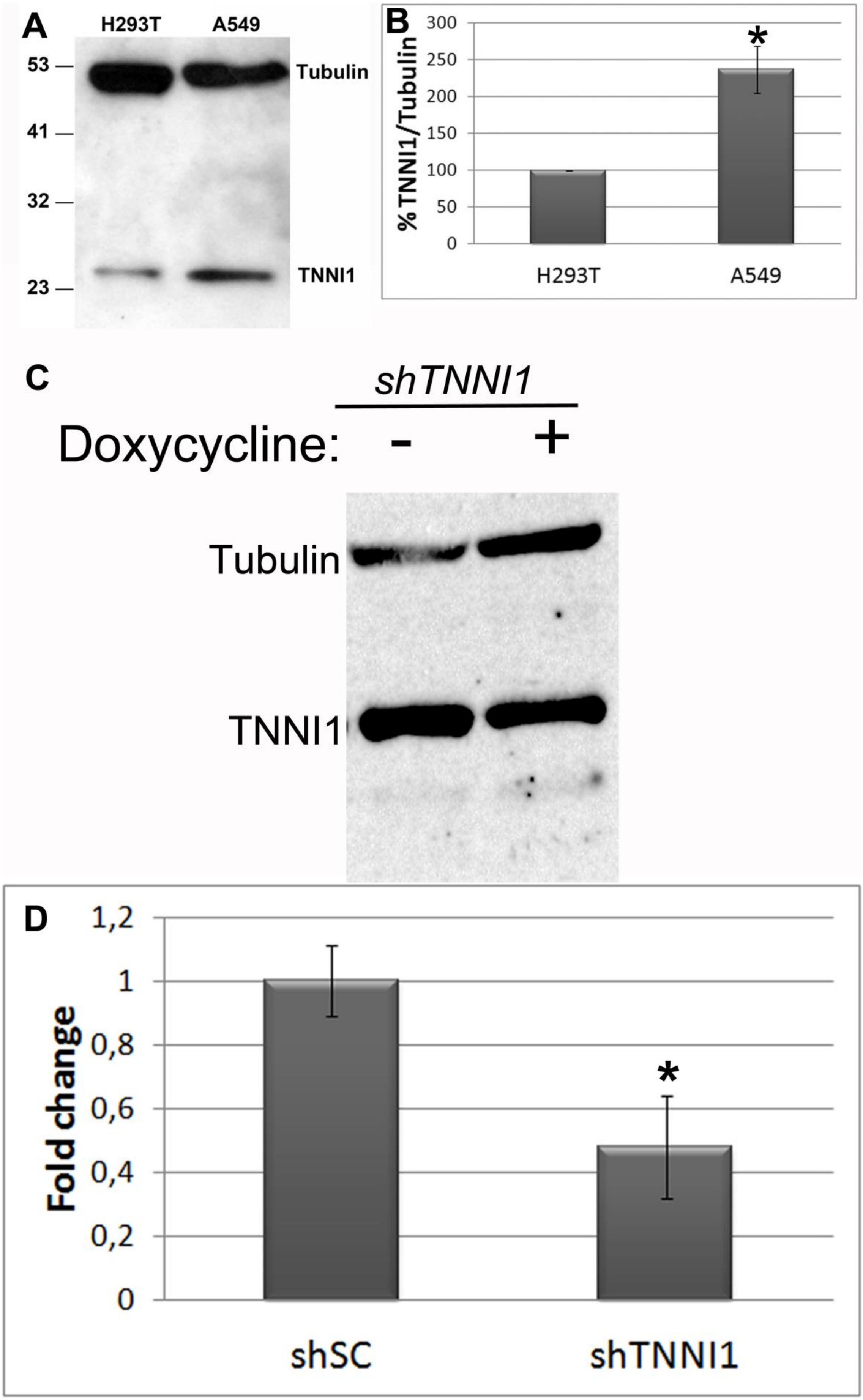
A
H293T
A549
53
41
32
23
Tubulin
TNNI1
B
% TNNI1/Tubulin
300
250
200
150
100
50
0
*
H293T
A549
C
shTNNI1
Doxycycline:
-
+
Tubulin
TNNI1
D
Fold change
1,2
1
0,8
0,6
0,4
0,2
0
*
shSC
shTNNI1

Supplementary Figure S6.- Human TNNI expression is efficiently reduced by sh*TNNI*. A) The tumor cell line A549, used in xenografts experiments, over-expresses TNNI1 in contrast to the control cell line H293T. **B)** Quantification of data shown in **A**. **C)** Analysis of TNNI1 expression in A549 cells transformed with an inducible short hairpin RNAi corresponding to a scrambled peptide sequence (*shSC*) as control, or an equivalent construct with the coding sequence of the gene (*shTNNI*). Cells were induced (+) or not (-) for 48 hours with 2 μM doxycycline. Arrow indicates an unspecific protein band. Note the reduction in TNNI1 levels in the *shTNNI*1+ lane. **D)** Quantification of TNNI1 protein versus Tubulin after inducing RNAi expression.

1. Nakato H, Futch TA and Selleck SB. The division abnormally delayed (dally) gene: a putative integral membrane proteoglycan required for cell division patterning during postembryonic development of the nervous system in Drosophila. Development. 1995; 121(11):3687-3702.
2. Gonzalez S, Klatt P, Delgado S, Conde E, Lopez-Rios F, Sanchez-Cespedes M, Mendez J, Antequera F and Serrano M. Oncogenic activity of Cdc6 through repression of the INK4/ARF locus. Nature. 2006; 440(7084):702-706.
3. Abengozar MA, de Frutos S, Ferreiro S, Soriano J, Perez-Martinez M, Olmeda D, Marenchino M, Canamero M, Ortega S, Megias D, Rodriguez A and Martinez-Torrecuadrada JL. Blocking ephrinB2 with highly specific antibodies inhibits angiogenesis, lymphangiogenesis, and tumor growth. Blood. 2012; 119(19):4565-4576.
